# Vitamin D3 stimulates embryonic stem cells but inhibits migration and growth of ovarian cancer and teratocarcinoma cell lines

**DOI:** 10.1186/s13048-016-0235-x

**Published:** 2016-04-18

**Authors:** Ahmed Abdelbaset-Ismail, Daniel Pedziwiatr, Ewa Suszyńska, Sylwia Sluczanowska-Glabowska, Gabriela Schneider, Sham S. Kakar, Mariusz Z. Ratajczak

**Affiliations:** Stem Cell Institute at James Graham Brown Cancer Center, University of Louisville, 500 S. Floyd Street, Rm. 107, Louisville, KY 40202 USA; Department of Physiology Pomeranian Medical University, Szczecin, Poland; Department of Regenerative Medicine Medical University of Warsaw, Warsaw, Poland

**Keywords:** Vitamin D3, VDR, Germline tumors, Ovarian cancer, Teratocarcinoma, VSELs

## Abstract

**Background:**

Deficiency in Vitamin D3 (cholecalciferol) may predispose to some malignancies, including gonadal tumors and in experimental models vitamin D3 has been proven to inhibit the growth of cancer cells. To learn more about the potential role of vitamin D3 in cancerogenesis, we evaluated the expression and functionality of the vitamin D receptor (VDR) and its role in metastasis of ovarian cancer cells and of murine and human teratocarcinoma cell lines.

**Methods:**

In our studies we employed murine embrynic stem cells (ESD3), murine (P19) and human (NTERA-2) teratocarcimona cells lines, human ovarian cancer cells (A2780) as well as purified murine and human purified very small embryonic like stem cells (VSELs). We evaluated expression of Vitamin D3 receptor (VDR) in these cells as well as effect of vitamin D3 exposure on cell proliferation and migration.

**Results:**

We here provide also more evidence for the role of vitamin D3 in germline-derived malignancies, and this evidence supports the proposal that vitamin D3 treatment inhibits growth and metastatic potential of several germline-derived malignancies. We also found that the ESD3 murine immortalized embryonic stem cell line and normal, pluripotent, germline-marker-positive very small embryonic-like stem cells (VSELs) isolated from adult tissues are stimulated by vitamin D3, which suggests that vitamin D3 affects the earliest stages of embryogenesis.

**Conclusions:**

We found that however all normal and malignant germ-line derived cells express functional VDR, Vitamin D3 differently affects their proliferation and migration. We postulate that while Vitamin D3 as anticancer drug inhibits proliferation of malignant cells, it may protect normal stem cells that play an important role in development and tissue/organ regeneration.

**Electronic supplementary material:**

The online version of this article (doi:10.1186/s13048-016-0235-x) contains supplementary material, which is available to authorized users.

## Background

Germline cell tumors are derived from cells endowed with early developmental potential. These tumors are located mostly in ovaries and testes, but they may also develop in the pelvis, in the mediastinal area, and intracranially [1]. It has been proposed that these extra-gonadal tumors arise from mutated primordial germ cells (PGCs) that migrate during embryogenesis and stray from their main route through the embryo proper to the genital ridges [2]. This theory of origin explains the most common locations of germline tumors along the PGC migratory route, beginning from the extraembryonic mesoderm, moving through the primitive streak, and ending in the developing gonads. The most notable of this type of extra-gonadal germline tumor is the sacrococcygeal teratoma, the single most common tumor diagnosed in babies at birth [3, 4]. This site marks the anatomical area where PGCs, after leaving the extraembryonic mesoderm, enter the embryo proper.

It has also been proposed that early in embryogenesis, stem cells expressing several germline characteristics are deposited in developing organs [5, 6]. These stem cells deposited at somatic sites may serve as a backup population for tissue-committed stem cells. Interestingly, very small embryonic-like stem cells (VSELs), which express several markers of migratory PGCs, have been identified in several organs, including bone marrow, ovaries, and testes [6, 7]. Moreover, cells with germline potential have been identified not only in gonads but also in bone marrow (BM), skin, esophagus, and even breast milk [8–10]. Similarly, exposure of BM mononuclear cells (MNCs) to chemical carcinogens revealed germline potential implicit in BM-adherent cells [11].

These examples are mostly in line with the so-called “embryonic rest hypothesis of cancer development”. During the 19th and early 20th centuries, it was proposed by Recamier (1829), Remak (1854), and Virchow (1858) and later elaborated by Durante (1874) and Cohnheim (1875) that cancer may originate in populations of cells that are left in a dormant state in developing organs during embryogenesis [12–16]. It is possible that certain developing tumors classified as germline tumors originate in such cells (stray migrating PGCs or VSELs). On the other hand, since several malignancies express so-called cancer testis antigens and respond to stimulation by pituitary and gonadal sex hormones [17, 18], we cannot exclude the possibility that other types of malignancies also originate in cells related to the germline [19, 20].

Vitamin D3 is a prohormone that prevents the development of tumors [21–25]. Specifically, a deficiency of this vitamin is implied in the origin of several malignancies, as it has been demonstrated in several experimental models that the growth of many tumor cell lines, including lung, breast, testicular, and ovarian cancer, as well as leukemia and lymphoma are inhibited by exposure to vitamin D3 [26, 27].

To better address the role of vitamin D3 in germline-derived tumors, we investigated the expression and potential role of the vitamin D3 receptor (VDR) in ovarian cancer and teratocarcinoma cells. We also evaluated the expression of the VDR in murine and human VSELs. Thus, our results provide more evidence for the potential role of vitamin D3 in germline-derived malignancies and support the proposal that vitamin D3 treatment inhibits the growth and metastatic potential of germline-derived malignancies [21, 22, 24, 25, 28]. Since we also found VDR expression on ESD3 murine embryonic stem cells, this observation supports the role of the VDR at the earliest stages of embryogenesis. Finally, we observed differences in the effect of vitamin D3 in malignant versus normal germline-related cells, which supports its having pleiotropic effects, both in physiological and pathological processes.

## Methods

### Cell lines

Both human (NTERA-2 teratocarcinoma, A2780 ovarian cancer) and murine (P19 embryonal teratocarcinoma) germline-derived immortalized cell lines were employed in our studies. All cell lines were purchased from American Type Culture Collection (ATCC; Manassas, VA, USA). NTERA-2 cells were cultured in Dulbecco’s modified Eagle medium (DMEM; GE Healthcare) with heat-inactivated 10 % fetal bovine serum (FBS; Seradigm). A2780 ovarian cancer cells were maintained in Roswell Park Memorial Institute (RPMI) medium 1640 containing L-glutamine (GE Healthcare) and 10 % heat-inactivated FBS. P19 embryonal carcinoma cells were cultured in minimum essential medium (MEM)-α (GE Healthcare) supplemented with ribonucleosides, deoxyribonucleosides, 2.5 FBS, and 7.5 % bovine calf serum. To all media, penicillin (100 units/mL; Corning) and streptomycin (10 *μ*g/mL; Corning) were added. All cells were cultured in a humidified atmosphere of 5 % CO_2_ at 37 °C, with exchange of medium every 48 h.

### Cultivation of the ESD3 murine embryonic stem cell line

The ESD3 cell line was purchased from ATCC and cultured in DMEM (GE Healthcare) with 4 mM L-glutamine, 1.5 g/L sodium bicarbonate, and 4.5 g/L glucose and supplemented with 0.1 mM 2-mercaptoethanol (Sigma-Aldrich), heat-inactivated 15 % FBS, and murine mature leukemia inhibitory factor (LIF, 5 ng/mL; Santa Cruz Biotechnology). Cells were maintained in a humidified atmosphere of 5 % CO_2_ at 37 °C, and the medium was exchanged every 48 h.

### Isolation of murine hematopoietic stem cells (HSCs) and very small embryonic-like stem cells (VSELs) from bone marrow

Bone marrow mononuclear cells (BMMNCs) were obtained from total BM cells flushed from femurs and tibias, lysed in BD lysing buffer (BD Biosciences, San Jose, CA) for 10 min at room temperature, washed twice in RPMI 1640 medium containing L-glutamine (Corning), and supplemented with 2 % heat-inactivated FBS. Next, MNCs were stained with the following antibodies: anti-CD45R/B220 (phycoerythrin [PE], clone RA-6B2), anti-Gr-1 (PE, clone RB6-8 C5), anti-T cell receptor αβ (PE, clone H57-5970), anti-T cell receptor ɤδ (PE, clone GL3), anti-CD11b (PE, clone M1/70), anti-Ter119 (PE, clone TER-119), anti-CD45 (allophycocyanin [APC]–Cy7, clone 30-F11), and anti-Ly-6A/E (also known as Sca-1, PE–Cy5 or Alexa Fluor 647, clone E13–161.7) for 30 min on ice. Cells were washed and resuspended in RPMI medium with 2 % FBS. Hematopoietic stem cells (HSCs; Sca-1^+^Lin^−^CD45^+^) and very small embryonic-like stem cells (VSELs; Sca-1^+^Lin^−^CD45^−^) were purified from MNCs using a multi-parameter Moflo XDP cell sorter (Beckman Coulter) as described [29].

### Isolation of human UCB-derived HSPCs and VSELs

Clinical-grade human umbilical cord blood (hUCB) research units obtained from Cleveland Cord Blood Center were used for isolation of hUCB-HSPCs and hUCB-VSELs as described in detail in our previous studies [30]. In brief, using a cocktail of biotin-conjugated monoclonal antibodies and anti-biotin monoclonal antibodies conjugated to paramagnetic microbeads (Lineage Cell Depletion kit, Miltenyi Biotec), magnetic labeling of retrieved total nucleated cells was performed, and the lineage-negative (Lin^−^) cells were isolated by depletion of mature hematopoietic cells expressing a panel of lineage antigens using an autoMACS separator (Miltenyi Biotec). Afterwards, the Lin^−^ population were stained with the following antibodies: anti-CD45 (PE or V450, clone HI30) and anti-CD34 (APC or PE, clone 581). After washing, the fluorochrome-labelled cells were resuspended and sorted to obtain populations enriched in HSCs (Lin^−^/CD45^+^/CD34^+^) and VSELs (Lin^−^/CD45^−^/CD34^+^).

### Reverse transcriptase-polymerase chain reaction (RT-PCR)

Total RNA was extracted and purified from teratocarcinomas and ESD3 cells using the RNeasy Mini kit (Qiagen Inc.) after treatment with DNase I (Qiagen Inc.) on a column. For VSELs and HSCs, total RNA was extracted using Trizol Reagent (Ambion; Austin, TX, USA) as described earlier [31]. The purified mRNA was afterwards reverse-transcribed into cDNA using Taqman Reverse Transcription Reagents (Applied Biosystems). Amplification of synthesized cDNA fragments was carried out using Amplitaq Gold polymerase (Applied Biosystems). The PCR conditions were: 1 cycle of 8 min at 95 °C; 2 cycles of 2 min at 95 °C, 1 min at 60 °C, and 1 min at 72 °C; 40 cycles of 30 s at 95 °C, 1 min at 60 °C, and 1 min at 72 °C; and 1 cycle of 10 min at 72 °C. Semi-nested PCR was performed only to evaluate expression of the VDR in sorted VSELs. The human and murine sequence-specific primers used for VDR amplification are listed in Additional file 1: Figure S1. Samples without template controls and reverse transcriptase were used in each run. All primers were designed using the NCBI/Primer-Blast program, as at least one primer included an exon–intron boundary. Afterwards, all PCR products were analyzed by 2 % agarose gel electrophoresis.

### Reverse transcription quantitative PCR (RT-qPCR)

RT-qPCR experiments were performed to detect and quantify relative levels of VDR mRNA in both human and murine germline-derived immortalized cell lines. The purified RNA was reverse-transcribed with MultiScribe Reverse Transcriptase, oligo(dT), and a random hexamer primer mix (all from Applied Biosystems Life Technologies, CA, USA). Quantitative evaluation of the target gene was then performed by using an ABI Prism 7500 sequence detection system (Applied Biosystems Life Technologies) with Power SYBR-green PCR Master Mix reagent and specific primers (Additional file 1: Figure S1). The PCR cycling conditions were 95 °C (15 s), 40 cycles at 95 °C (15 s), and 60 °C (1 min). According to melting point analysis, only one PCR product was amplified under these conditions. The relative quantity of a target gene, normalized to the β2-microglobulin gene as the endogenous control and relative to a calibrator, was expressed as 2^–ΔΔCt^ (fold difference), where Ct is the threshold cycle, ΔCt = (Ct of target genes) – (Ct of the endogenous control gene, β2-microglobulin), and ΔΔCt = (ΔCt for target gene in test sample) – (ΔCt for target gene in calibrator sample).

### In vivo BrdU labelling studies with murine HSCs and VSELs

Normal 2-month-old C57Bl6 mice (male) were exposed to a once-daily oral dosage of 8 × 10^2^ IU 1,25-dihydroxyvitamin D3 in sesame oil for five consecutive days. Control mice received vehicle orally. Mice were also exposed to BrdU (1 mg/animal/100 μL; BD Pharmingen) 2 days before starting with 1,25-dihydroxyvitamin D3 treatment and continued to the end of the experiment as described [29]. Control mice were injected with saline and BrdU solution. After 30 days, mice were sacrificed, and a single-cell suspension was obtained and stained for the HSC and VSEL phenotypes as described above. After cell-surface staining of cells, the FITC BrdU Flow kit (BD Pharmingen) staining protocol was employed, including fixation and permeabilization, treatment with DNase to expose incorporated BrdU, and finally staining with anti-BrdU–FITC antibody. After washing, samples were analyzed using a BD LSR II flow cytometer (BD Biosciences). At least one million events were captured and analyzed using BD FACSDiva software.

### Transwell migration assay

All cell lines were rendered quiescent by incubation in their basic medium supplemented with 0.5 % bovine serum albumin (BSA, Sigma-Aldrich) at 37 °C and then seeded at a density of 6 × 10^4^ cells/100 μL/insert into the upper chambers of Transwell inserts with 8-*μ*m polycarbonate membranes (Corning). The lower Boyden chambers received 1,25-dihydroxyvitamin D3 (Sigma-Aldrich) at both physiological (10^−10^ M) and supra-physiological (10^−9^–10^−7^ M) concentrations in culture medium with 0.5 % BSA. The lower chambers containing 10 FBS and 0.5 % BSA (plus vehicle) in RPMI 1640 medium served as a positive and negative control, respectively. After 24-h stimulation at 37 °C, the upper chambers were carefully removed, the cells that had not migrated were removed with a cotton applicator swab from the upper side, and the cells that had transmigrated to the lower side of the membrane were fixed and stained with HEMA-3 reagent (Protocol, Fisher Scientific, Pittsburgh, PA) and then counted using an inverted microscope.

### Adhesion of malignant cells to fibronectin

All cell lines were made quiescent for 3 h with 0.5 % BSA in basic medium in a humidified atmosphere of 5 % CO_2_ at 37 °C. Next, the cells were stimulated with 1,25-dihydroxyvitamin D3 (at concentrations ranging from 10^−10^–10^−7^ M), SDF-1 (300 ng/mL), or 0.5 % BSA (plus vehicle) in their respective basic culture media. Cells were then added directly and allowed to adhere to the fibronectin-coated wells (3000 cells/well) in 96-well plates at 37 °C. The wells were first coated with 70 *μ*L of fibronectin (Sigma-Aldrich; 10 *μ*g/mL) overnight at 4 °C and blocked before the experiment with BSA for 2 h at 37 °C. After a 5-min incubation at 37 °C, the wells were vigorously washed three times with PBS, and the remaining adherent cells were counted directly under an inverted microscope.

### Signal transduction studies

To evaluate the functionality of the vitamin D receptor (VDR), quiescent cells were stimulated with 0.5 % BSA medium (containing vehicle) or 1,25-dihydroxyvitamin D3 (10^−10^, 10^−9^, 10^−8^, or 10^−7^ M) for 5 min or 12 h at 37 °C. The harvested cells were washed with PBS, lysed with RIPA lysis buffer supplemented with protease and phosphatase inhibitors (Santa Cruz Biotech) for 30 min on ice, and centrifuged at 15,000 rpm at −4 °C for 15 min. The protein concentration was measured using the Pierce BCA Protein Assay Kit (Pierce, Rockford, IL). The adjusted extracted proteins (20 μg/each sample) were then separated on a 4–12 % SDS-PAGE gel, and the fractionated proteins were transferred to a PVDF membrane (Bio-Rad). All membranes were blocked with 2.5 % BSA in Tris-buffered saline containing 0.1 % Tween (TBST) for 1 h at room temperature. After washing with TBST, phosphorylation of the intracellular kinase p44⁄42 mitogen-activated protein kinase (p42/44 MAPK) and AKT was detected by incubating the membranes with phosphospecific anti-p-p42/44 MAPK (clone no. 9101, diluted 1:1000) and anti-p-AKT (Ser473; clone no. 9271, diluted 1:1000) rabbit polyclonal antibodies (Cell Signaling), respectively, overnight at 4 °C. Horseradish peroxidase (HRP)-conjugated goat anti-rabbit IgG was used as a secondary antibody (Santa Cruz Biotech, 1:5000) and was incubated with the PVDF membranes for 2 h at RT. To confirm equal protein loading in all lanes, blots were stripped using stripping buffer (Thermo Scientific) and then reprobed with appropriate anti-rabbit p42/44 MAPK (clone no. 9102) and anti-rabbit AKT (clone no. 9272) monoclonal antibodies (both from Cell Signaling). All membranes were treated with an enhanced chemiluminescence (ECL) reagent (Amersham Life Sciences) and subsequently exposed to film (Hyperfilm, Amersham Life Sciences). For band visualization, the automatic machine supplied with fresh warm developer and fixer solutions was used.

### Cell proliferation

Cells were cultured in 96-well plates (Cell Star; Greiner Bio-One) at an initial density of 3 × 10^3^ cells/mL with 0.5 % BSA in DMEM (NTERA2), RPMI 1640 (A2780), or αMEM (P19) medium in the presence or absence of 1,25-dihydroxyvitamin D3 (10^−10^ M, 10^−9^ M, 10^−8^ M, or 10^−7^ M). The medium containing 0.5 % BSA with vehicle was used as a negative control, while full medium containing 10 % FBS was treated as a positive control. The cell number was calculated directly after cell seeding (0 h) as well as at 24, 48, and 72 h after adding 1,25-dihydroxyvitamin D3. At these time points, cells were harvested from the wells after trypsinization and counted using FACS.

### Cell transplantation into immunodeficient mice

Prior to in vivo transplantation, human germline-derived immortalized cells were pretreated ex vivo with 1,25-dihydroxyvitamin D3 (10^−9^ M) or vehicle alone for 12 h. The cells were then washed and transplanted (10 × 10^5^ per mouse) into severe combined immunodeficient (SCID)-beige inbred mice (*n* = 3 per group), which were irradiated with 750 cGy 24 h before transplantation. At 48 h post transplantation, bone marrows, livers, and lungs were collected, and the presence of metastasized cancer cells (i.e., murine–human chimerism) was evaluated as described [32]. Briefly, genomic DNA was purified from organs using the QIAamp DNA Mini kit (Qiagen). Next, detection of human α-satellite and murine β-actin DNA levels was carried out using real-time PCR and the ABI Prism Fast 7500 Sequence Detection System (Applied Biosystems). A 25-*μ*L reaction mixture containing 12.5 *μ*L SYBR Green PCR Master Mix, 300 ng DNA template, and specific primers (5′-accactctgtgtccttcgttcg-3′ [sense] and 5′-actgcgctctcaaaaggagtgt-3′ [antisense] primers for α-satellite DNA; 5′-ttcaattccaacactgtcctgtct-3′ [sense] and 5′ ctgtggagtgactaaatggaaacc-3′ [antisense] primers for β-actin DNA) were used. Real-time PCR conditions for the amplification process were as follows: 95 °C (15 s); 40 cycles at 95 °C (15 s), and 60 °C (1 min). Samples without template controls were used in each run. The ΔCt values were determined, where Ct is the threshold cycle. For each cell line, the number of human cells present in the murine organs (the degree of chimerism) was calculated according to the standard curve generated by mixing different concentrations of human cells with a constant number of murine cells in a linear manner.

### Annexin V–phosphatidylserine binding assay

Human carcinoma cells were treated in RPMI/0.5 % BSA medium containing either 1,25-dihydroxyvitamin D3 (10^−10^ M, 10^−9^ M, or 10^−8^ M) or vehicle only. Cells subjected to culture medium with 10 % DMSO were used as a positive control. After a 12-h treatment period, the cells were gently detached using non-enzymatic cell dissociation buffer (Sigma-Aldrich), pelleted, and washed twice with cold PBS. Next, to define the levels of early and late apoptotic cells, the FITC Annexin V Apoptosis Detection kit (#556547; BD Pharmingen) was employed according to the manufacturer’s protocol. In brief, 1 × 10^5^ cells/100 μL Annexin V Binding Buffer were incubated in the dark with FITC–annexin V and propidium iodide (PI), as a vital dye, for 15 min at 25 °C. The cells that were left unstained or stained with either FITC–annexin V or PI served as compensation staining controls. Afterwards, cells were analyzed by flow cytometry after adding Annexin V Binding Buffer (400 *μ*L) to all tubes. The percentage of cells that had undergone apoptosis was then determined by calculating the percentage of apoptotic cells in the untreated population (vehicle) from the percentage of apoptotic cells in the 1,25-dihydroxyvitamin D3-treated population.

### Data analysis

Statistical analysis was carried out using GraphPad Prism 6 (GraphPad Software Inc) and Sigma software (Sigma Software Inc). All data are presented as mean ± SD. Statistical analysis of the data was done using one-way ANOVA and Tukey’s test for post hoc pairwise multiple comparison. In all analyses, *p* ≤ .05, *p* ≤ .01, and *p* ≤ .001 were considered significant.

## Results

### Functional vitamin D3 receptor (VDR) is expressed in established human and murine germline cell lines and in very small embryonic-like stem cells (VSELs)

To address the role of vitamin D3 in germline-derived cells, we first evaluated expression of the VDR receptor in human and murine germline-derived cells and next evaluated whether these receptors are functional by studying the effect of vitamin D3 stimulation on phosphorylation of MAPKp42/44 and AKT kinases.

**Fig. 1:**
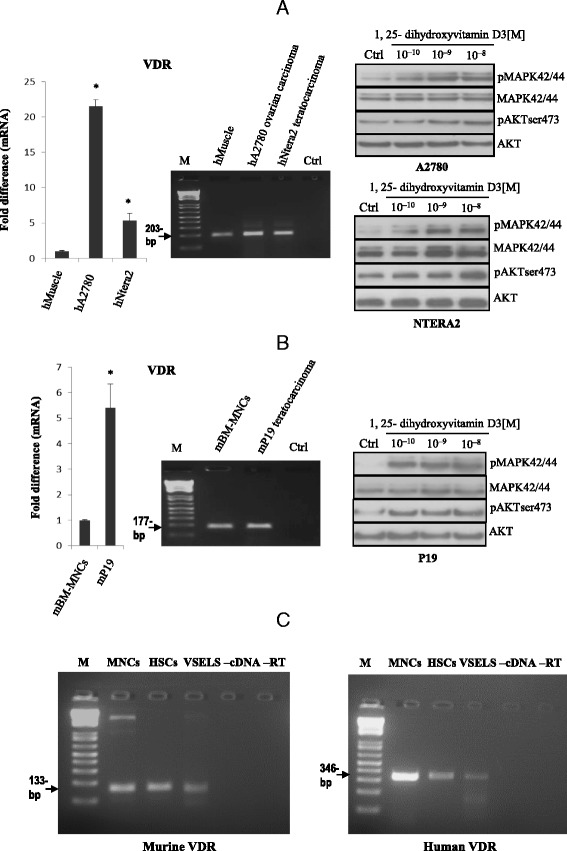
Human and murine germline-derived immortalized cell lines and FACS-sorted very small embryonic-like stem cells (VSELs) and hematopoietic stem cells (HSCs) express functional vitamin D receptors (VDRs). Expression of VDRs was detected in purified mRNA samples from human teratocarcinoma (hNTERA2) and ovarian cancer (hA2780) cell lines (Panel **a**, *left*) as well as from the murine embryonal teratocarcinoma (mP19) cell line (Panel **b**, *left*) by both real-time and conventional reverse transcription polymerase chain reaction (RT-PCR). The effect of 1,25-dihydroxyvitamin D3 on phosphorylation of p42/44 MAPK and AKT^ser473^ intracellular pathway proteins in hNTERA2 and hA2780 cell lines (Panel **a**, *right*) and the mP19 cell line (Panel **b**, *right*) was investigated. Cells (2 × 10^6^ cells/mL) were starved for 12 h in their respective basic culture media containing 0.5 % BSA and stimulated afterwards for 5 min with 1,25-dihydroxyvitamin D3 at various concentrations (10^−10^–10^−8^ M) or with vehicle (DMSO) only. The experiment was carried out twice with similar results, and representative blots are shown. Panel **c** The VDR is expressed by sorted VSELs and HSCs. RT-PCR showed expression of the VDR by both murine BM-derived (*left panel*) and human UCB-derived (*right panel*) VSELs, HSCs, and MNCs. In all experiments, samples with water only instead of cDNA (−cDNA) and without reverse transcriptase (−RT) were used as negative controls. Representative agarose gels of the RT-PCR amplicons obtained are shown. Each experiment was carried out twice with similar results, and representative blots are shown

Figure 1a, b, left panels show that the human ovarian cancer cell line (A2780) as well as the human (NTERA2) and murine (P190) teratocarcinoma cell lines express mRNA for the VDR. Importantly, we provide evidence that stimulation by vitamin D3 induces phosphorylation of MAPKp42/44 and AKTser473 in these cells (Fig. 1a, b, right panels). Next, we isolated mRNA from human and murine VSELs and the corresponding hematopoietic stem cells (HSCs) and detected expression of VDR mRNA in all these cells. As expected, the VDR was also expressed in peripheral blood mononuclear cells (MNCs, Fig. 1c, left and right panels).

To address whether VSELs respond to stimulation by vitamin D3, we applied it for a prolonged period of time in mice to evaluate the incorporation of bromodeoxyuridine (BrdU) into VSELs and HSCs. We found that, while ~30 % of murine HSCs from vitamin D3-treated mice were BrdU^+^ regardless of whether they were injected with vitamin D3, the number of quiescent BrdU^+^ VSELs increased from ~7 % to ~14 % in mice injected with vitamin D3 (Additional file 2: Figure S2).

This result supports the conclusion that functional VDRs are expressed on primitive human and murine germline-derived tumor cells as well as on normal VSELs.

### Vitamin D3 inhibits spontaneous migration, adhesion, and proliferation of germline-derived tumor cells

**Fig. 2:**
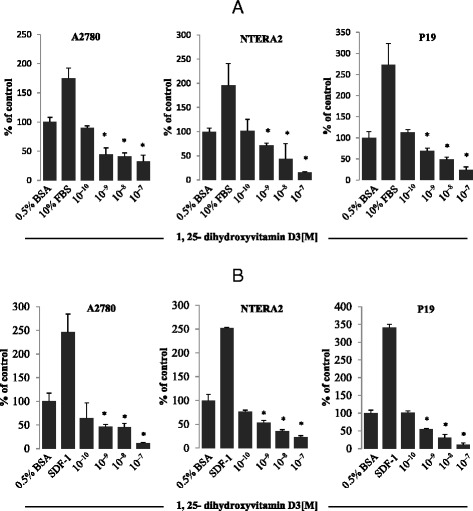
Panel **a** 1,25-dihydroxyvitamin D3 inhibits migration of human and murine germline-derived cell lines. Transmigration of a human ovarian cancer cell line (A2780), a human teratocarcinoma cell line (NTERA-2), and a murine embryonal teratocarcinoma cell line (P19) through Transwell membranes (8-μm pore size) in response to 1,25-dihydroxyvitamin D3 at the indicated concentrations. Cells were rendered quiescent in 0.5 % BSA in culture medium overnight at 37 °C. The effects of 1,25-dihydroxyvitamin D3 on migration of all cell lines employed (6 × 10^4^ cells/100 *μ*L/insert) were also evaluated in parallel for migration in response to 10 % FBS and 0.5 % BSA plus vehicle as a positive and negative control, respectively. Twenty-four hours later, the migrated cells were stained and counted using an inverted microscope. Panel **b** 1,25-dihydroxyvitamin D3 interferes with the adhesiveness of human and murine germline-derived cell lines to fibronectin. Adhesion of the A2780 human ovarian cancer cell line, the NTERA-2 human teratocarcinoma cell line, and the P19 murine embryonal teratocarcinoma cell line to fibronectin-coated surfaces in response to 1,25-dihydroxyvitamin D3. After three hours of quiescence, cells (3000 cells/100 *μ*L) were stimulated with 1,25-dihydroxyvitamin D3 at the indicated concentrations in medium with 0.5 % BSA for 5 min at 37 °C. After the non-adherent cells were removed by three consecutive washes with PBS, the number of adherent cells was measured by microscopic analysis. The effects of 1,25-dihydroxyvitamin D3 on adhesion of all cell lines employed were also evaluated for adhesion compared with stromal-derived factor 1 (300 ng/mL) and culture medium containing 0.5 % BSA plus vehicle as a positive and negative control, respectively. The negative control values are normalized to 100 %. Data are displayed as means ± SD, with a statistical significance *p ≤ 0.05 versus control (unstimulated) cells

Based on observations from the literature that vitamin D3 has anti-metastatic properties [23, 24], we evaluated spontaneous migration of human and murine germline-derived tumor cells in response to vitamin D3 and observed a dose-dependent decrease in their spontaneous migration (Fig. 2, panel a) as well as adhesion to fibronectin (Fig. 2, panel b).

**Fig. 3:**
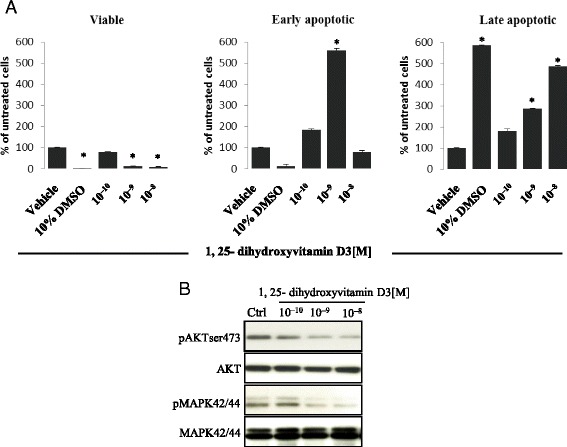
1,25-dihydroxyvitamin D3 induces apoptosis of human ovarian cancer cells in vitro. Panel **a** The A2780 human ovarian cancer cell line was treated with 1,25-dihydroxyvitamin D3 at concentrations ranging from 10^−10^–10^−8^ M for 16 h, and apoptosis was detected using the AnnexinV–FITC apoptosis kit and analyzed using flow cytometry. The data shown here indicate the induction of significant early apoptosis in cells treated with 10^−9^ M 1,25-dihydroxyvitamin D3 compared with cells treated with vehicle only. In this experiment, 10 % DMSO was used as positive control. The means of two experiments in triplicate were used. For statistical comparisons, a one-way analysis of variance and a Tukey’s test for post hoc analysis were carried out, and means ± SD are shown. Significance level: *p ≤ 0.05 versus control (untreated) cells. Panel **b** In parallel, treatment of the A2780 human ovarian cancer cell line with 1,25-dihydroxyvitamin D3 led to inhibition of phosphorylation of the p42/44 MAPK and AKT^ser473^ intracellular pathway proteins in dose-dependent responses. Human ovarian cancer cells (2 × 10^6^ cells/mL) were incubated for 12 h in RPMI with 0.5 % BSA culture medium containing either 1,25-dihydroxyvitamin D3 at various concentrations (10^−10^–10^−8^ M) or vehicle only. The experiment was carried out twice with similar results, and representative blots are shown

These changes in migration and adhesion can be explained by the pro-apoptotic effects of increasing doses of vitamin D3 (Fig. 3). As shown in Fig. 3, panel a, exposure of the A2780 ovarian cancer cell line to vitamin D3 resulted in an increase in the number of early and late apoptotic cells, which correlated with impaired vitamin D3 signaling in these cells (Fig. 3, panel b).

**Fig. 4:**
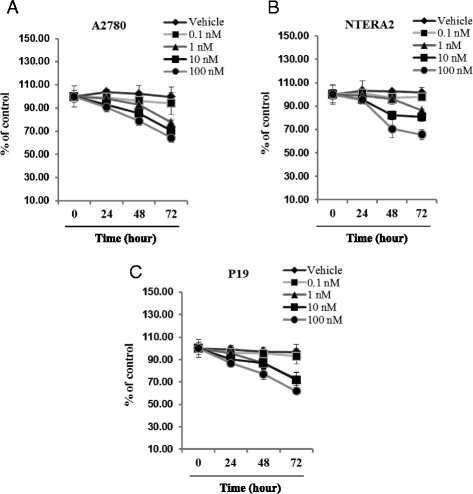
1,25-dihydroxyvitamin D3 suppresses proliferation of human and murine germline-derived cell lines. Proliferation of the A2780 human ovarian cancer cell line (Panel **a**) as well as the NTERA-2 human teratocarcinoma (Panel **b**) and mP19 murine embryonal teratocarcinoma (Panel **c**) cell lines was significantly decreased by 1,25-dihydroxyvitamin D3 in a dose-dependent manner in comparison with cells treated with vehicle only. All proliferation experiments were done either in RPMI (for A2780), DMEM (for NTERA-2), or αMEM (for P19) culture medium containing 0.5 % BSA for 72 h using 0.3 × 10^4^ cells/well in a 96-well plate. The negative control values are normalized to 100 %. For each cell line, the experiment was repeated twice in triplicate with similar results

In parallel, these apoptotic changes corresponded with an inhibitory effect of vitamin D3 on proliferation of human and murine germline-derived malignant cell lines (Fig. 4).

### Exposure of germline-derived malignant cells to vitamin D3 inhibits their seeding efficiency after injection into immunodeficient mice

**Fig. 5:**
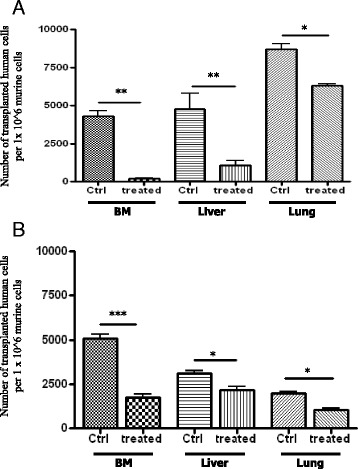
1,25-dihydroxyvitamin D3 inhibits metastasis of human ovarian cancer and teratocarcinoma cells. Detection of in vivo-transplanted 1,25-dihydroxyvitamin D3-treated A2780 human ovarian cancer cells (**a**) and hNTERA-2 human teratocarcinoma cells (**b**) in the organs of irradiated mice post transplantation. As shown, the number of 1,25-dihydroxyvitamin D3-treated cancer cells was significantly lower in isolated organs from mice than in ex vivo-untreated cells (vehicle only). Detection was performed by employing RT-qPCR for the presence of human Alu sequences in purified genomic DNA samples. For statistical comparisons, a one-way analysis of variance and a Tukey’s test for post hoc analysis were carried out, and means ± SD are shown. Significance levels: *p ≤ 0.05, **p ≤ 0.01, ***p ≤ 0.001 versus control (untreated) cells

Next, to assess the effect of vitamin D3 on the metastatic potential of germ line-derived tumor cells in vivo, we exposed A2780 human ovarian cancer (Fig. 5, panel a) and NTERA2 human teratocarcinoma cells (Fig. 5, panel b) to vitamin D3 before i.v. injection into immunodeficient mice. Incubation of tumor cells before injection with vitamin D3 decreased seeding efficiency of these cells into bone marrow, liver, and lung. This decrease in seeding efficiency can again be explained by the combined pro-apoptotic (Fig. 3), anti-proliferative (Fig. 4), anti-migratory (Fig. 2, panel a), and anti-adhesive (Fig. 2, panel b) effects of vitamin D3.

### The ESD3 murine embryonic stem cell line expresses functional VDRs

Finally, to address how early in embryonic development functional VDRs are expressed, we evaluated expression of this receptor on cells from the ESD3 murine embryonic cell line and observed that this receptor is expressed and responds to stimulation by vitamin D3 by phosphorylation of p42/44 MAPK and AKT (Additional file 3: Figure S3). Interestingly, in contrast to malignant cells derived from germline tumors, we found that vitamin D3 does not inhibit migration of these cells.

## Discussion

The salient observation of the current work is that functional vitamin D3 receptors (VDRs) are expressed in a broad range of normal and malignant cells whose origin is related to the germline. In addition, it is known that the VDR is not only expressed by ovarian and testicular cancer cells [22, 28] but is also expressed on murine and human teratocarcinoma cells, murine embryonic stem cells, and developmentally early stem cells that express several primordial germ cell markers isolated from adult tissues, which have been described as very small embryonic-like stem cells (VSELs) [5–7, 31].

Vitamin D3 plays an important role in several physiological and pathological processes [26, 33]. It ameliorates hypertension, prevents osteoporosis, shows anti-diabetic effects, and plays an important role in embryonic development, including gametogenesis [34–38]. In the female reproductive system, vitamin D3 is involved in several stages of folliculogenesis, as it is essential in cell proliferation, cell differentiation, and estrogen biosynthesis [39]. These positive effects are partially explained by attenuation of the negative effects of TGF-β1 on gametogenesis [38]. The role of vitamin D3 is also well documented in the male reproduction system. Specifically, mutant VDR-null mice develop reduced fertility later in life, which implies that the effect of vitamin D3 is exerted directly on the germ cells and/or indirectly by modulating Leydig cell function [38].

Evidence has accumulated from epidemiological studies that vitamin D3 and exposure to solar ultraviolet radiation have inverse relationships with non-skin cancer mortality. Specifically, sunlight protects against ovarian, breast, prostate, and colon cancer [40]. On the other hand, vitamin D3 deficiency enhances the risk of several malignancies, including gonadal tumors [41]. Consistent with this effect, multiple ovarian and testicular cancer cell lines respond to stimulation by vitamin D3 by growth suppression and induction of apoptosis [22], and the antiproliferative effect of this prohormone is mediated by increasing p27 protein stability and up-regulation of GADD45 [42, 43]. On the other hand, induction of apoptosis in ovarian cancer cell lines has been explained by downregulation of mRNA stability of the telomerase catalytic subunit [44]. Vitamin D3 or its analogs have been demonstrated to inhibit the growth of ovarian cancer tumors in in vivo xenograft animal models and inhibit angiogenesis in those malignancies [23]. Vitamin D3 has also been demonstrated to show synergistic anti-tumor effects in combination with cytostatics such as carboplatin [45]. Interestingly vitamin D3 has been reported to inhibit leptin-mediated cancer growth by upregulating miR-498, which downregulates leptin-enhanced expression of mRNA encoding telomerase reverse transcriptase (hTERT) [46]. Finally, decreased expression of CYP27B1, which hydroxylates 25-hydroxyvitamin D3 into biologically active 1,25-dihydroxyvitamin D3 (calcitriol) correlates with the increased pro-metastatic potential of ovarian cancer cells [21].

In our work we have shown that, like other ovarian cancer cell lines, human A2780 expresses functional VDRs and that its growth is inhibited by vitamin D3 in a dose-dependent manner. We observed a parallel effect on the viability of these cells. Moreover, we present new results that ovarian cancer cells exposed to vitamin D3 show impaired seeding efficiency in vivo to BM, liver, and lung after intravenous injection into immunodeficient mice.

Importantly, in addition to a negative effect on ovarian cancer cell growth, we report here that vitamin D3 also inhibits proliferation of human and murine teratocarcinoma cell lines. In similar sets of experiments, we found that these cells express functional VDRs and that vitamin D3 inhibits their growth in vitro and ameliorates metastatic potential in vivo after injection into immunodeficient mice. Functional VDRs were also found to be expressed by murine immortalized embryonic stem cells. Thus, expression of VDRs on these cells suggests why the developing embryo is susceptible to changes in vitamin D3 exposure [47].

Interestingly, in contrast to established germline-derived cell lines, we found that vitamin D3 stimulates proliferation of normal developmentally early stem cells residing in adult tissues (VSELs). We observed that prolonged administration of vitamin D3 to mice resulted in an increase in BrdU incorporation into these otherwise very quiescent cells residing in bone marrow (BM) [48]. This is interesting for several reasons. First, VSELs have been reported to be at the top of the mesenchymal lineage hierarchy and are thus precursors for mesenchymal stem cells [49, 50]. Based on our results, the increase in the number of VSELs may at least partially explain the beneficial effect of vitamin D3 supplementation on bone growth and in ameliorating or preventing osteoporosis [35]. It is most likely that, by giving rise to mesenchymal stem cells, VSELs supply new osteoblasts, cartilage cells, and stromal cells.

It has also been reported in several elegant papers that VSEL-like cells reside in adult ovaries and testes [51, 52]. In parallel, it has been proposed that gonadal VSELs could be the precursors of gametes [53–55]. Thus, it is possible that some of the abovementioned beneficial effects of vitamin D3 on the reproductive system can be explained by the direct effect of vitamin D3 on the gonadal population of VSELs.

The expression of VDRs in germline-derived cells as well as VSELs provides additional support for a developmental relationship between these cells [49]. We have already demonstrated that VSELs express several markers of migrating primordial germ cells [7]. Moreover, our recent work demonstrated expression of functional erythropoietin receptors on germline-derived cells, including ovarian cancer and teratocarcinoma cells and VSELs [56]. Expression of functional VDRs again supports a potential developmental relationship between these cells.

In conclusion, our report shows that the VDR is expressed across a spectrum of germline-derived tumors, from teratocarcinoma cells to ovarian cancer cells, and supports our proposal that vitamin D3 is an inhibitor of their proliferation. We also provide new evidence that normal germline-related stem cells (VSELs) can proliferate in vivo after administration of vitamin D3. This dual (and opposing) role of vitamin D3 on proliferation of normal germline-derived cells and inhibition of malignant germline tumor growth explains better some beneficial effects of vitamin D3 supplementation in normal as well as in pathological processes.

## Conclusions

Based on this data vitamin D3 supplementation is important for normal development and embryogenesis as it promotes growth of normal development early stem cells, and on other hand we provide further justification to employ vitamin D3 in the clinic as an anticancer compound to inhibit tumor expansion.

### Ethics approval and consent to participate

Animal study reported was approved by Institutional Animal Care and Use Committee (IACUC), University of Louisville.

### Availability of data and materials

The dataset supporting the conclusion of this article is included within the article and 23 its additional files.

## Additional files

Additional file 1: Figure S1. The list and sequences of the primer pairs used in this study. (PPT 142 kb) Additional file 2: Figure S2. 1,25-dihydroxyvitamin D3 induces proliferation of quiescent BM-derived very small embryonic-like stem cells (VSELs) in vivo. Panel A. After treatment with 1,25-dihydroxyvitamin D3, as shown here, no significant difference in BrdU-incorporated HSCs was observed in treated and control animals (6 male mice/group). Panel B. BrdU incorporation into VSELs after 1,25-dihydroxyvitamin D3 treatment. The percentages of VSELs that incorporated BrdU into newly synthesized DNA. After treatment with 1,25-dihydroxyvitamin D3 at 800 IU/dose, ~13.2 % of VSELs were BrdU^+^, in contrast to the control group in which ~7 % of VSELs were BrdU^+^ (6 male mice/group). The incorporation of BrdU into HSCs and VSELs was measured by FACS. (PPT 100 kb) Additional file 3: Figure S3. ESD3 murine embryonic stem cells express functional VDRs. Expression of VDRs was detected in purified mRNA samples from the ESD3 cell line as well as in murine BM-derived HSCs and MNCs (Panel A) by conventional reverse transcription polymerase chain reaction (RT-PCR). Samples with water only instead of cDNA (−cDNA) and without reverse transcriptase (−RT) were used as negative controls. Representative agarose gels of the RT-PCR amplicons obtained are shown. The effect of 1,25-dihydroxyvitamin D3 on phosphorylation of p42/44 MAPK and AKT^ser473^ intracellular pathway proteins in ESD3 cells (Panel B) was assessed. Cells (10^6^ cells/mL) were starved for 12 h in their respective culture media containing 0.5 % BSA in an incubator and afterwards stimulated for 5 min with 1,25-dihydroxyvitamin D3 at various concentrations (0.1–100 nM) or with vehicle (DMSO) only. The experiment was carried out twice with similar results, and representative blots are shown. Panel C. Vitamin D3 does not inhibit Transwell migration of murine ESD3 cells. The experiment was performed twice with similar results. (PPT 293 kb)

## Abbreviations

ANOVA: analysis of variance
ATCC: American Type Culture Collection
BM: bone marrow
BMMNCs: bone marrow mononuclear cells
BNCs: mononuclear cells
BrdU: 5-Bromo-2′-deoxyuridine
BSA: bovine serum albumin
DMEM: Dulbecco’s modified Eagle medium
DMSO: dimethyl sulfoxide
ECL: enhanced chemiluminescence
FACS: fluorescence-activated cell sorting
FBS: fetal bovine serum
FITC: fluorescein isothiocyanate
HSCs: hematopoietic stem cells
hUCB: human umbilical cord blood
LIF: leukemia inhibitory factor
MEM: minimum essential medium
PGCs: primordial germ cells
RPMI: Roswell Park Memorial Institute
RT-PCR: reverse transcriptase-polymerase chain reaction
SCID: severe combined immunodeficiency
SD: standard deviation
SDS-PAGE: sodium dodecyl sulfate-Polyacrylamide gel electrophoresis
VDR: vitamin D receptor
VSELs: very small embryonic like cells
