# Supplementary material for: Vitamin D3 stimulates embryonic stem cells but inhibits migration and growth of ovarian cancer and teratocarcinoma cell lines
Source: J Ovarian Res. 2016 Apr 18;9:26. doi: 10.1186/s13048-016-0235-x (PMC4835879; doi:10.1186/s13048-016-0235-x)
Supplement: Additional file 1: Figure S1. — The list and sequences of the primer pairs used in this study. (PPT 142 kb) [file 13048_2016_235_MOESM1_ESM.ppt]

## Slide 1
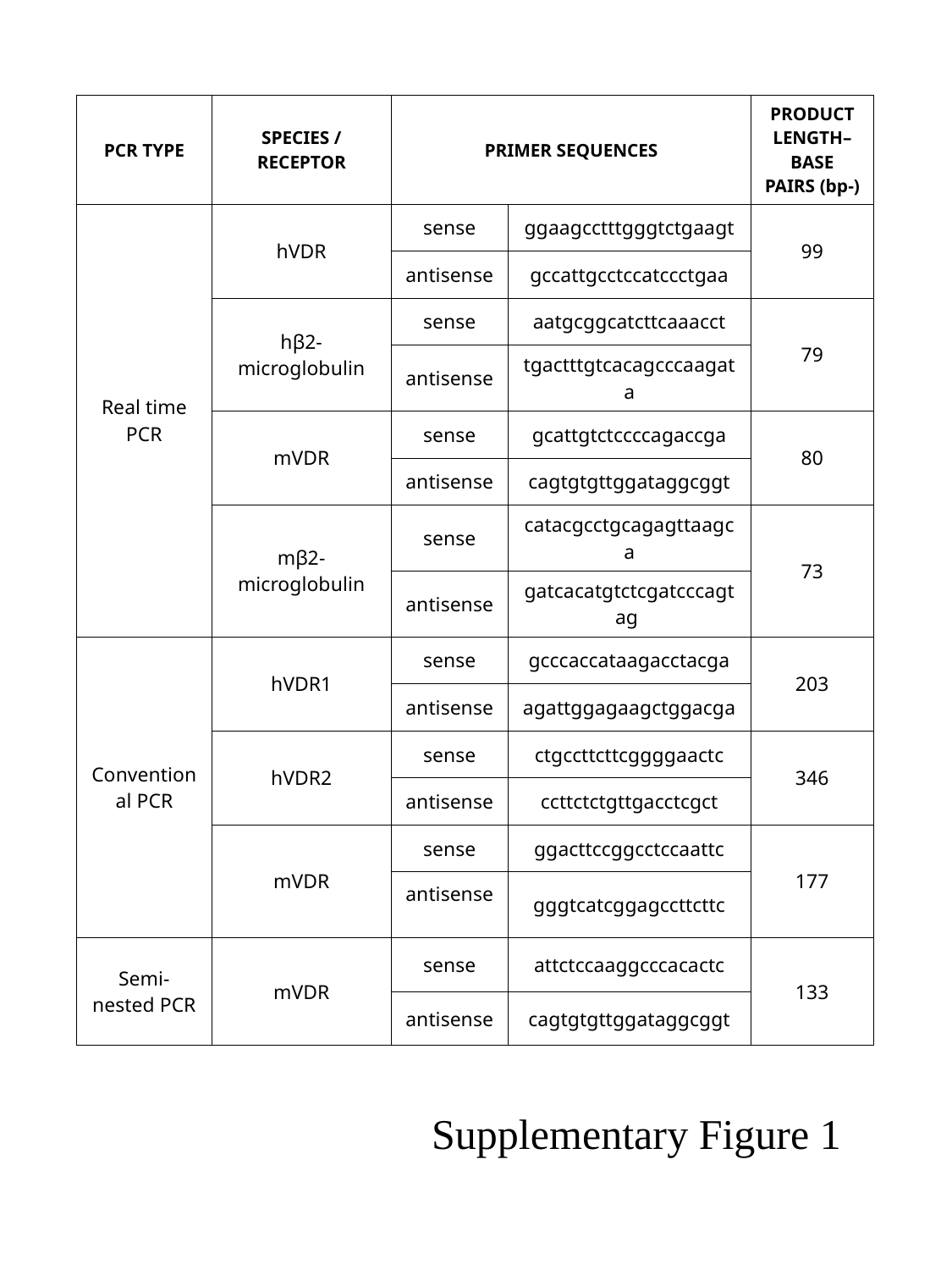

| PCR TYPE | SPECIES / RECEPTOR | PRIMER SEQUENCES | | PRODUCT LENGTH–BASE PAIRS (bp-) |
| --- | --- | --- | --- | --- |
| Real time PCR | hVDR | sense | ggaagcctttgggtctgaagt | 99 |
| | | antisense | gccattgcctccatccctgaa | |
| | hβ2-microglobulin | sense | aatgcggcatcttcaaacct | 79 |
| | | antisense | tgactttgtcacagcccaagata | |
| | mVDR | sense | gcattgtctccccagaccga | 80 |
| | | antisense | cagtgtgttggataggcggt | |
| | mβ2-microglobulin | sense | catacgcctgcagagttaagca | 73 |
| | | antisense | gatcacatgtctcgatcccagtag | |
| Conventional PCR | hVDR1 | sense | gcccaccataagacctacga | 203 |
| | | antisense | agattggagaagctggacga | |
| | hVDR2 | sense | ctgccttcttcggggaactc | 346 |
| | | antisense | ccttctctgttgacctcgct | |
| | mVDR | sense | ggacttccggcctccaattc | 177 |
| | | antisense | gggtcatcggagccttcttc | |
| Semi-nested PCR | mVDR | sense | attctccaaggcccacactc | 133 |
| | | antisense | cagtgtgttggataggcggt | |
Supplementary Figure 1
