# Supplementary figures and images for: Vitamin D3 stimulates embryonic stem cells but inhibits migration and growth of ovarian cancer and teratocarcinoma cell lines
Source: J Ovarian Res. 2016 Apr 18;9:26. doi: 10.1186/s13048-016-0235-x (PMC4835879; doi:10.1186/s13048-016-0235-x)

## Slide 1
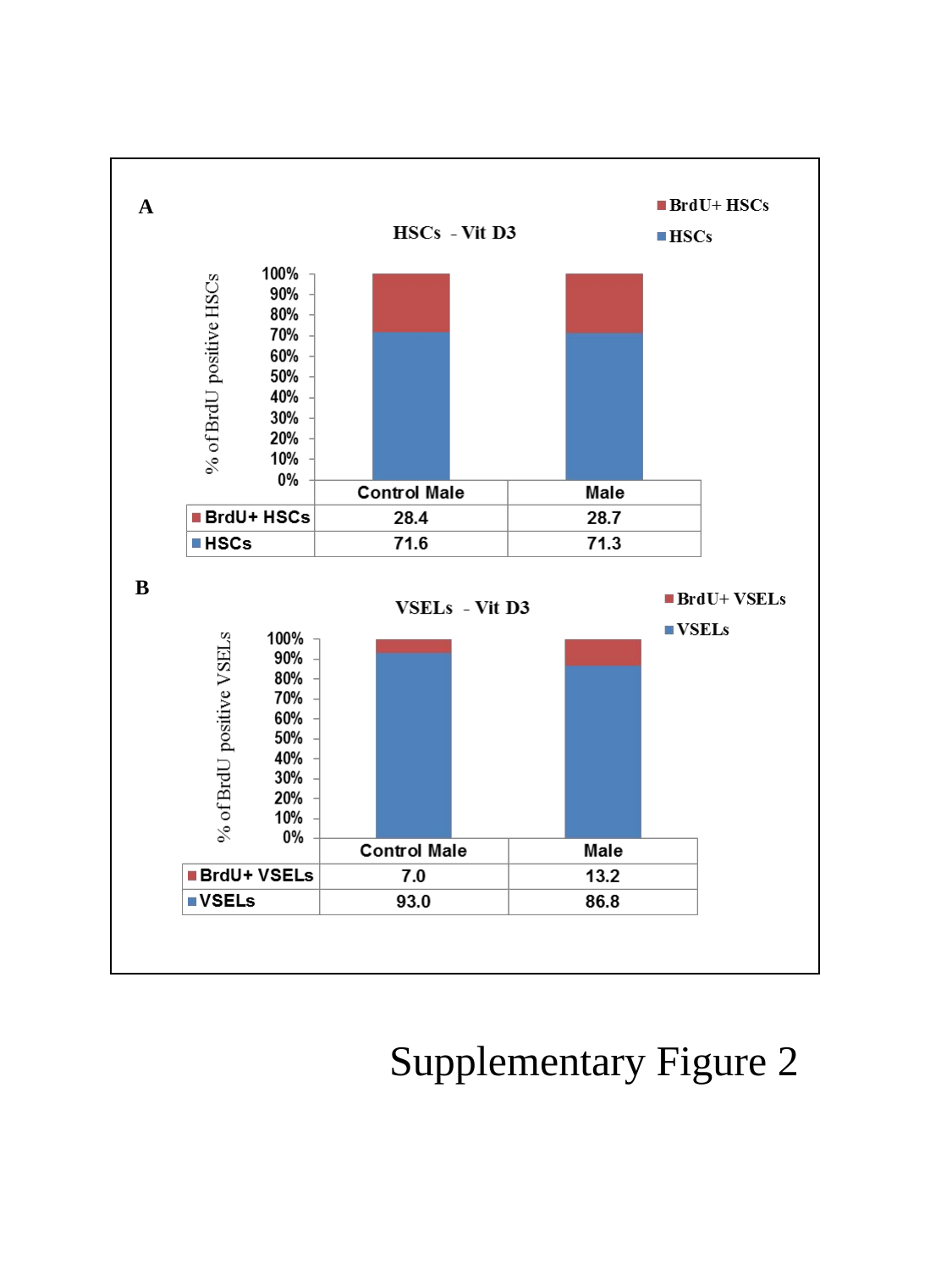

A
B
Supplementary Figure 2

Supplement: Additional file 2: Figure S2. — 1,25-dihydroxyvitamin D3 induces proliferation of quiescent BM-derived very small embryonic-like stem cells (VSELs) in vivo. Panel A. After treatment with 1,25-dihydroxyvitamin D3, as shown here, no significant difference in BrdU-incorporated HSCs was observed in treated and control animals (6 male mice/group). Panel B. BrdU incorporation into VSELs after 1,25-dihydroxyvitamin D3 treatment. The percentages of VSELs that incorporated BrdU into newly synthesized DNA. After treatment with 1,25-dihydroxyvitamin D3 at 800 IU/dose, ~13.2 % of VSELs were BrdU+, in contrast to the control group in which ~7 % of VSELs were BrdU+ (6 male mice/group). The incorporation of BrdU into HSCs and VSELs was measured by FACS. (PPT 100 kb) [file 13048_2016_235_MOESM2_ESM.ppt]
