# Supplementary material for: Vitamin D3 stimulates embryonic stem cells but inhibits migration and growth of ovarian cancer and teratocarcinoma cell lines
Source: J Ovarian Res. 2016 Apr 18;9:26. doi: 10.1186/s13048-016-0235-x (PMC4835879; doi:10.1186/s13048-016-0235-x)
Supplement: Additional file 3: Figure S3. — ESD3 murine embryonic stem cells express functional VDRs. Expression of VDRs was detected in purified mRNA samples from the ESD3 cell line as well as in murine BM-derived HSCs and MNCs (Panel A) by conventional reverse transcription polymerase chain reaction (RT-PCR). Samples with water only instead of cDNA (−cDNA) and without reverse transcriptase (−RT) were used as negative controls. Representative agarose gels of the RT-PCR amplicons obtained are shown. The effect of 1,25-dihydroxyvitamin D3 on phosphorylation of p42/44 MAPK and AKTser473 intracellular pathway proteins in ESD3 cells (Panel B) was assessed. Cells (106 cells/mL) were starved for 12 h in their respective culture media containing 0.5 % BSA in an incubator and afterwards stimulated for 5 min with 1,25-dihydroxyvitamin D3 at various concentrations (0.1–100 nM) or with vehicle (DMSO) only. The experiment was carried out twice with similar results, and representative blots are shown. Panel C. Vitamin D3 does not inhibit Transwell migration of murine ESD3 cells. The experiment was performed twice with similar results. (PPT 293 kb) [file 13048_2016_235_MOESM3_ESM.ppt]

## Slide 1
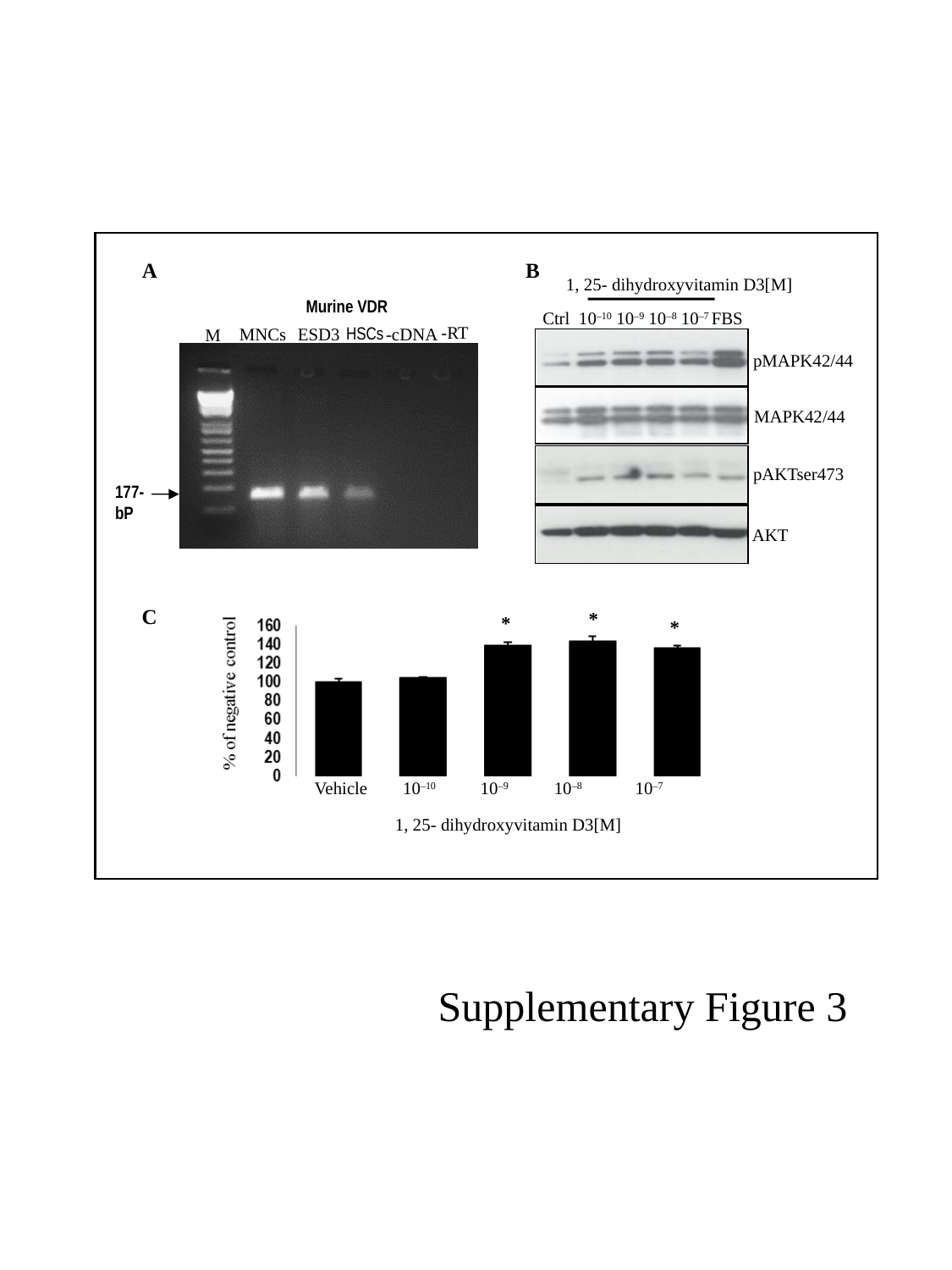

A
B
1, 25- dihydroxyvitamin D3[M]
pMAPK42/44
MAPK42/44
pAKTser473
AKT
Ctrl 10‒10 10‒9 10‒8 10‒7 FBS
Murine VDR
-RT
HSCs
MNCs
ESD3
-cDNA
M
177-
bP
*
*
*
Vehicle 10‒10 10‒9 10‒8 10‒7
1, 25- dihydroxyvitamin D3[M]
C
Supplementary Figure 3
